# Accurate, comprehensive database of group I introns and their homing endonucleases

**DOI:** 10.1093/bioadv/vbaf020

**Published:** 2025-02-05

**Authors:** Lara Sellés Vidal, Tomoya Noma, Yohei Yokobayashi

**Affiliations:** Nucleic Acid Chemistry and Engineering Unit, Okinawa Institute of Science and Technology Graduate University, Onna, Okinawa 9040495, Japan; Nucleic Acid Chemistry and Engineering Unit, Okinawa Institute of Science and Technology Graduate University, Onna, Okinawa 9040495, Japan; Nucleic Acid Chemistry and Engineering Unit, Okinawa Institute of Science and Technology Graduate University, Onna, Okinawa 9040495, Japan

## Abstract

**Motivation:**

Group I introns are one of the most widely studied ribozymes. Since their initial discovery, a large number of them have been identified experimentally or computationally. However, no comprehensive and unified database that provides group I intron sequences with precise boundaries and structural information is available.

**Results:**

We created a new database of group I intron sequences with reliable exon-intron boundaries. The database offers additional data for each sequence, such as containing GenBank entry, its position within the associated entry, the subtype of each intron and putative homing endonucleases. Secondary structure predictions and base-pairing probability matrixes are also provided for each sequence. The resource is expected to facilitate large-scale studies of group I introns, as well as engineering for novel applications.

**Availability and implementation:**

The database, as well as the code to generate it and a GUI to facilitate its exploration, are available at https://github.com/LaraSellesVidal/Group1IntronDatabase. The source code for the GUI implementation is available at https://github.com/LaraSellesVidal/OnlineGroup1IntronDatabase. The database can also be accessed online at https://online-group-1-intron-database.onrender.com. Base-pairing probability matrixes are available separately at https://www.ebi.ac.uk/biostudies/studies/S-BSST1399.

## 1 Introduction

Group I introns are self-splicing RNA sequences that catalyze their own excision through a series of transesterification reactions without help from protein cofactors (Haugen *et al.* 2005). Since their original discovery in 1982, group I introns have been identified in all domains of life. They have been found in a variety of genes in bacterial, viral, and archaeal genomes (Yamada *et al.* 1994, Hausner *et al.* 2014, Nawrocki *et al.* 2018), in the ribosomal DNA of eukaryotes (Hedberg and Johansen 2013), and in mitochondrial and plastid DNA of a variety of organisms, including some multicellular eukaryotes (Kuhsel *et al.* 1990, Huchon *et al.* 2015).

Group I introns are of high interest for multiple reasons. First, the parental sequences from which group I introns originated are thought to be ancient, pre-biotic RNA molecules, making these introns possible relics of the hypothetical RNA world (Lehman 2009, Raghavan and Minnick 2009). Their study could therefore bring valuable insights into the origin of complex catalytic activities required for life. Additionally, their self-splicing ability has been exploited for a variety of applications. For example, extensive engineering and modifications of the group I introns based on the mechanistic understanding have led to trans-splicing introns for sensing and RNA editing (Dolan and Müller 2014), and RNA circularization for RNA stabilization (Lee *et al.* 2023).

Such studies would greatly benefit from the availability of a comprehensive resource listing a large number of known group I intron sequences with precise boundaries and their properties. However, the existing databases for group I introns do not offer precise intron boundaries for many sequences. For example, the Group I Intron Sequence and Structure Database (GISSD) (Zhou *et al.* 2008) only offers precise boundaries and surrounding exonic context for a reduced subset of the sequences, with sequences in the comprehensive dataset lacking essential elements such as P9.

Here, we present a novel, up-to-date resource that addresses some of the shortcomings of the available resources to provide a solid foundation for evolutionary and engineering studies of group I introns. The presented database offers 42 459 intron sequences with precise boundaries, exonic context for each intron, and rich metadata (including subtype classification and the host gene and organism). Additionally, we also annotate the presence or absence of homing endonucleases (HEGs) in each intron, and where applicable, provide its sequence. Finally, we provide secondary structure predictions and base-pairing probability matrixes for all sequences obtained with multiple state-of-the-art methods. The latter are provided as separate files, connected with the central intron list through the use of shared intron IDs.

## 2 Previous resources

Collections of group I introns can be found in both specialized and general RNA databases. The only previous example of a specialized database collecting group I introns is GISSD. Nevertheless, as previously mentioned, it suffers from shortcomings such as lacking precise intron boundaries. Furthermore, as of July 2024, the resource is partially inaccessible. Regarding general RNA databases, both Rfam (Kalvari *et al.* 2021) and the Comparative RNA Web (CRW) (Cannone *et al.* 2002) include group I intron sequences. However, in both cases the number of listed sequences is limited (2611 in Rfam and 201 in CRW), and lacks key annotations (for example, Rfam introns are not classified by subtypes, and while CRW provides the organism for each intron, a classification into broader phylogenetic groups is not given). Importantly, none of the previously described resources provides associated HEGs identified within the provided intron sequences. We therefore aimed to provide an updated resource overcoming the described limitations of previous databases. Table 1 summarizes the features of the different collections of group I introns, including our work.

**Table 1.** Comparison of group I intron resources.

	GISSD^a^	CRW	Rfam	This work
Intron no.	1789/20 085	201	2611	42 459
Subtype classification	Yes	Yes	No	Yes
Structure predictions	Covariation-based	Covariation-based	Covariation-based	Covariation-based, other six methods
Base-pairing probability matrices	No	No	No	Yes
Metadata annotations	Species, subcellular location, and subtype	Species and subcellular location	Species	Species, subcellular location, and subtype
Intron boundaries determination	Infernal hit only	GenBank annotations only	Infernal hit only	Both Infernal hit and GenBank annotations
Homing endonucleases	No	No	No	Yes

a Not fully accessible as of September 2024.

## 3 Methods and features

In order to obtain a comprehensive database of group I introns, we started by running an Infernal 1.1.5 (Nawrocki and Eddy 2013) search of the covariance model (CM) of group I introns available in Rfam against the entire NCBI NT database (February 2024 release) (National Library of Medicine (US), National Center for Biotechnology Information 2024b) (*cmsearch --nohmmonly -E 1000 -Z 1300000 --tblout output_file cm_file NT_fasta_file*). This led to a total of 245 292 hits constituting candidate group I introns, which were used as the starting point to generate the current version of the presented resource. Our first goal was to determine new and accurate boundaries for each of the candidate introns identified through Infernal. To do so, we first extracted the associated GenBank (National Library of Medicine (US), National Center for Biotechnology Information 2024a) entry ID and boundaries of the Infernal hit. The list of annotated sequence features (genetic elements of biological significance) of the GenBank entry was then iterated over, and the candidates for new intron boundaries were extracted as follows:

- If a feature is of type intron, and its annotated boundaries define a range that overlaps by at least 50 positions with the range of the Infernal hit, it is added to a list of candidate new intron boundaries. This allows to extract candidate intron boundaries from sequence regions that have been already annotated as introns.
- If a feature has a compound location, defined by the union of multiple ranges, we iterate over all consecutive pairs of parts. If any pair of parts is found such that the gap between the end of the first part and the beginning of the second part overlaps by at least 50 positions with the range of the Infernal hit, it is also added to the list of candidate new intron boundaries. This enables identification of intron boundaries from regions that are not explicitly annotated as introns, but are located between regions that are annotated as exons of the same gene (e.g. through transcriptomics analysis).
- After iterating over all features of the corresponding GenBank entry for a given Infernal hit, if there are multiple candidate new intron boundaries, the candidate with the largest overlap with the range of the Infernal hit is selected.

For each Infernal hit for which new intron boundaries were identified through this procedure, the following additional conditions were imposed to confirm the validity of the newly determined intron boundaries:

- The beginning of the new intron range must not be the first position of the corresponding GenBank entry
- The end of the new intron range must not be the last position of the corresponding GenBank entry. These two conditions ensure that the flanking exonic context is known.
- The subsequence defined by the new boundaries must contain a U at its initial position (the last nucleotide of the preceding exon). This condition was imposed because this U is involved in the formation of a completely conserved catalytic U-G pair required for intron cleavage.

The above procedure leads to the identification of accurate intron boundaries for a total of 42 459 of the introns contained in the starting list of Infernal hits (16 427 were rejected due to not starting with a U), which constitute the current version of our group I intron database. Each intron entry was then extended by adding the following pieces of data:

- Exonic context: the sequences of the preceding and following exons (up to 150 nucleotides for each) were extracted from the corresponding GenBank entries.
- Corresponding species.
- Category of organism: the species of each group I intron were classified using full taxonomies retrieved from NCBI’s Taxonomy database (National Library of Medicine (US), National Center for Biotechnology Information 2024d).
- Subcellular location (when applicable): for eukaryotic cells, we specify if the intron is located in the nucleus, mitochondria, or plastids.

We then classified the introns into previously described subtypes (Michel and Westhof 1990, Li and Zhang 2005). In order to do so, we first used Infernal to construct CMs for each subtype from previously described multiple alignments of sequences belonging to each subtype (Zhou *et al.* 2008), and a CM database was assembled from these. Then, a search of each sequence against the CM database was performed (*cmscan –max --tblout output_cmscan_file cm_database_file intron_fasta_file*). Each intron was classified into the subtype giving the hit with the highest score, and aligned to the individual CM of the corresponding subtype (*cmalign –ileaved --mxsize 900000 --nonbanded -o output_alignment_file subtype_CM_file intron_fasta_file*). Alignment information was used together with the consensus secondary structure element of each subtype to annotate structural elements for each intron. To further enrich our database, we also calculated secondary structure predictions and base-pairing probability matrixes for all introns with ViennaRNA (Lorenz *et al.* 2011), NUPACK (Fornace *et al.* 2022), RNAstructure (Reuter and Mathews 2010), RNAsoft (Andronescu *et al.* 2003), CONTRAfold (Do *et al.* 2006), and EternaFold (Wayment-Steele *et al.* 2022). Secondary structures based on Infernal alignments and all other secondary structure predictions are provided in the main database file (Appendix 1 lists and describes all its fields), and base-pairing probability matrixes are provided as separate files for each intron.

Next, we aimed to annotate the HEGs potentially present in the introns. Group I introns are known to occasionally contain an open reading frame (ORF) encoding a HEG able to recognize and cleave highly specific DNA sequences, which leads to the integration of group I introns in the cleavage point as a consequence of homology-driven DNA repair (Stoddard 2014). Identification of ORFs encoding HEGs was performed as follows:

1. For each species, the applicable genetic codes for all its subcellular locations were obtained from the NCBI Taxonomy database (National Library of Medicine (US), National Center for Biotechnology Information 2024c).
2. The intron was translated into all possible six reading frames using all known genetic codes.
3. Candidate ORFs with a minimum length of 120 amino acids were selected, taking into consideration the valid start and stop codons of each genetic code. For nested ORFs, only the largest one was kept.
4. InterProScan (Jones *et al.* 2014) was run for all found candidate ORFs. Briefly, InterProScan integrates multiple resources that provide information about protein function, such as assignment of the family to which a protein belongs to, or identification of domains, motifs and sites with particular function or structural features. InterProScan was run with default parameters, which performs all possible analyses in the run.
5. For each intron, ORFs returning at least one hit corresponding to a HEG family were annotated as putative HEGs. Such hits were identified by selecting those that contained at least one substring associated with HEGs (*endonuc*, *homing*, or *nuclease*) in any of its annotations. The previously retrieved genetic codes applicable to each organism and subcellular location were used to annotate for each putative HEG if it originates from an ORF with the expected genetic code, with a genetic code expected for the corresponding organism but from a different subcellular location, or from a genetic code not used by that organism.
6. For the remaining introns with candidate ORFs but no HEG hit through InterProScan, a DELTA-BLAST search of all its candidate ORFs was performed against the NCBI NR database (National Library of Medicine (US), National Center for Biotechnology Information 2024b). DELTA-BLAST was run with default parameters and using the RPSDB database as the conserved domains database (CDD). ORFs producing hits against regions of sequences annotated as endonucleases were also included as putative HEGs of the containing intron. The DELTA-BLAST origin of these HEGs was also annotated, including the NR entry that led to the hit and genetic code information as in the case of InterProScan hits.

This led to the identification of putative homing endonucleases in 6900 introns. The boundaries, reading frame, sequence and source of annotation as a HEG of each are also provided in the database.

Both the main intron database file, as well as the database of identified HEGs, are provided as tab-separated value (TSV) files. This facilitates direct manipulation with tools such as Python, R, and AWK while enabling direct access through standard spreadsheet editors such as Microsoft Excel (see Appendix 2 for details) or macOS Numbers. Details about the implementation of the database can be found on the GitHub repository (https://github.com/LaraSellesVidal/Group1IntronDatabase) in file *full_workflow_clean.ipynb*, a commented Jupyter notebook reproducing and explaining all steps taken to create the resource.

The database of introns can also be browsed through a graphical user interface (GUI), implemented in the form of a Python *Flask* application and available both online at https://online-group-1-intron-database.onrender.com and as a standalone desktop packaged with the *flaskwebgui* library. The source code for the GUI implementation is also available at https://github.com/LaraSellesVidal/OnlineGroup1IntronDatabase.

## 4 Results and discussion

**Figure 1.**
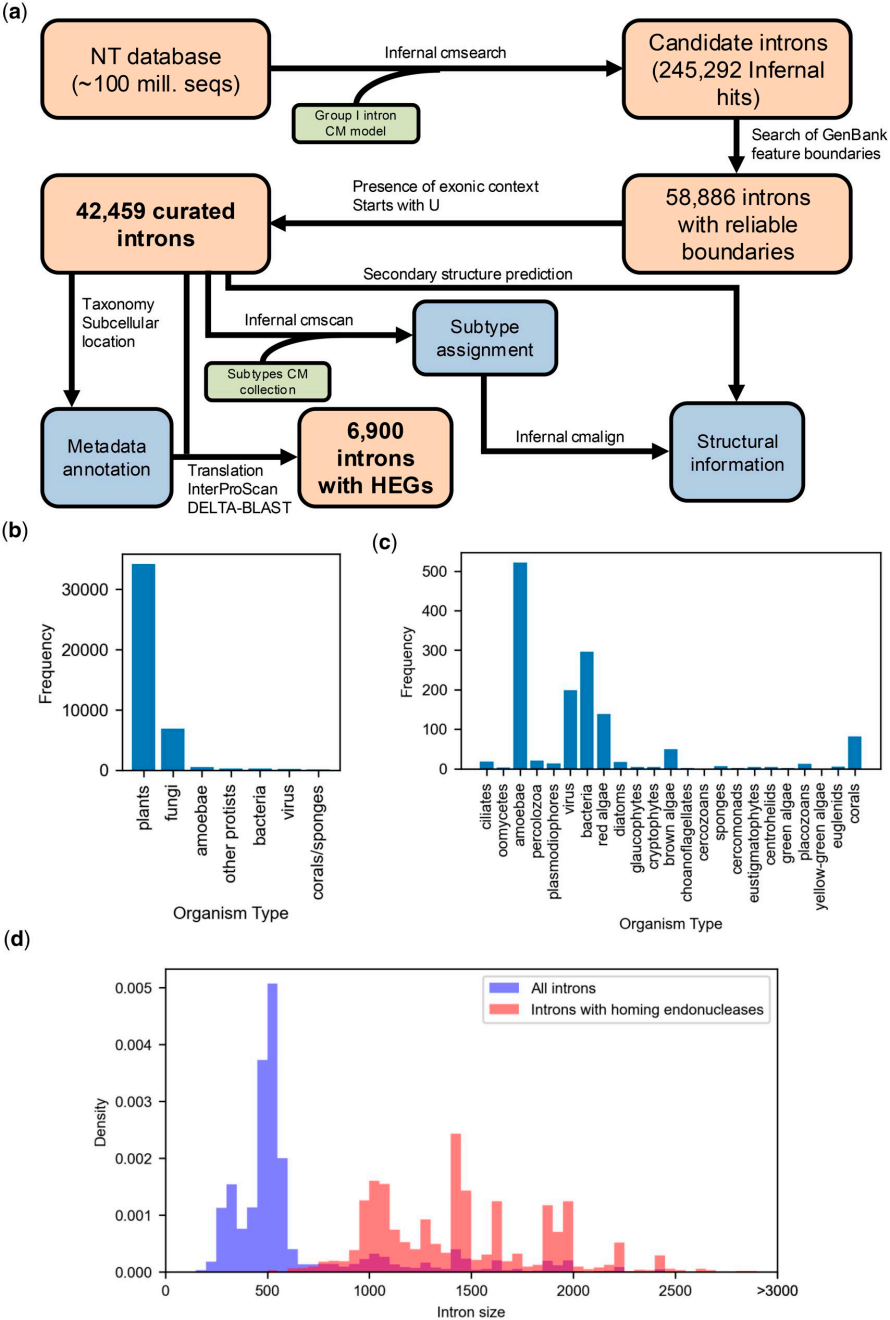
A comprehensive database of group I introns. (a) Workflow followed to generate the database of group I intron with reliable boundaries and identify putative homing endonucleases in them. The workflow was applied to the entire NT database. (b) Bar plot showing the distribution of identified group I introns across organism types. The majority of group I introns were found in plants, followed by fungi. (c) As in (b), but excluding plants and fungi. (d) Comparison of the length distribution of all introns and only of introns with homing endonucleases. As expected, the latter are larger.

By following the described workflow (Fig. 1a), we were able to obtain a set of 42 459 group I introns with well-defined boundaries. The resulting resource greatly facilitates statistical analysis of this type of introns. We obtained a distribution of group I introns by category of organisms (Fig. 1b and c), which revealed that the majority of group I introns are present in plants. This is in accordance with the fact that group I introns are widespread in tRNA genes of chloroplasts of higher plants (Kuhsel *et al.* 1990). The second most frequent group of organisms were fungi, with nearly 7000 introns found in organisms of this category. Fungal nuclear ribosomal RNA genes are indeed known to frequently contain group I introns (Bhattacharya *et al.* 2005).

**Figure 2.**
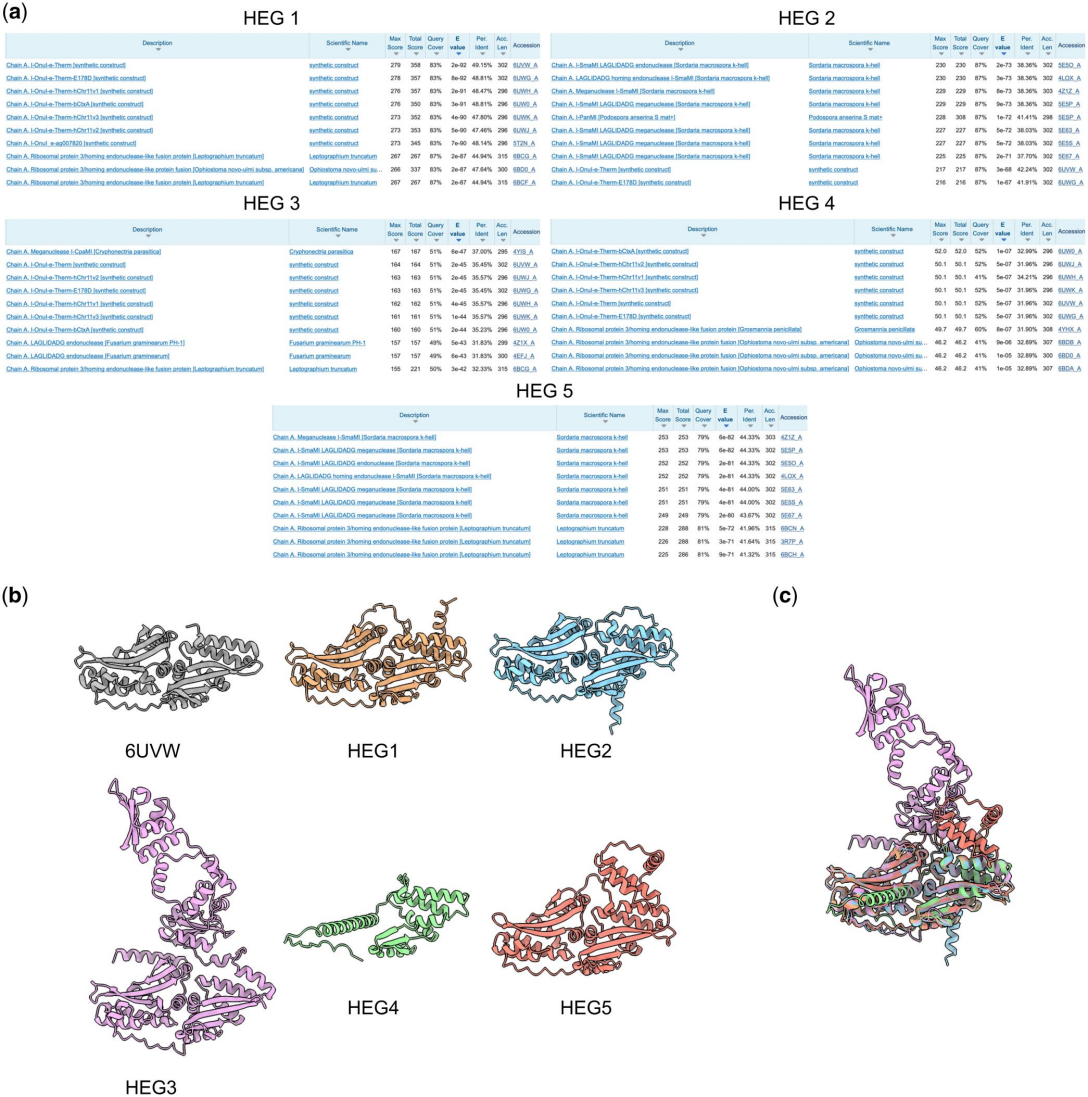
Validation of multiple homing endonucleases found in a single intron. (a) BLAST hits obtained with each of the five homing endonucleases (HEGs) identified in intron NC_062883.1_4. BLAST was performed only against sequences with an experimental structure deposited in the PDB. Only the top 10 hits are shown for each sequence. In all cases, significant hits against homing endonucleases were found. (b) Comparison of the structure of a HEG of the double-motif LAGLIDADG family (chain A of PDB ID 6UVW; grey) with the structures predicted by AlphaFold 3 for the 5 HEGs identified in intron NC_062883.1_4. In all cases except HEG4, the predicted structures contain all the standard fold of a double-motif LAGLIDADG HEG, and differ in the insertions present in flexible loops. The structure predicted for HEG4 corresponds instead to that of a single-motif LAGLIDADG HEG. (c), Overimposition of the predicted structures for the five HEGs of intron NC_062883.1_4.

We also identified putative HEGs in a total of 6900 group I introns. Importantly, the search for putative HEGs was performed not only with the genetic code expected for each intron according to its containing organism and subcellular location. Instead, all possible genetic codes were used during candidate ORF detection, and the match/mismatch with the expected genetic code was included for each putative HEG as metadata. Interestingly, while the majority of HEGs were identified with the expected genetic code (5581 HEGs in 5084 introns), we also found a set of introns (1816) for which, while no putative HEG was detected from candidate ORFs of the expected genetic code, they were indeed found using other genetic codes. The presence of these suggests that they were acquired through lateral gene transfer from a different subcellular location or organism (where the genetic code was indeed appropriate to allow expression of the contained HEG), Alternatively, it is possible that mutations in the original sequence led to the HEG not being anymore expressed in the expected genetic code, remaining as a relic within its containing intron. As expected, the size distribution of introns with and without homing endonucleases reveals a shift to longer sequences in introns with putative homing endonucleases (Fig. 1d). Surprisingly, we found some occurrences of introns with multiple, non-overlapping putative homing endonucleases. The highest number (five) was found in an intron of the gene encoding cytochrome b in the mitochondrial genome of *Exserohilum turcicum* (GenBank ID NC_062883.1; intronID NC_062883.1_4), a phytopathogenic fungus (Ma *et al.* 2022). To our knowledge, no previous reports of group I introns containing multiple homing endonucleases are available. Interestingly, two of the putative homing endonucleases are encoded in different reading frames and overlap by 25 nucleotides. In order to further validate the five identified HEGs, their sequences were subjected to BLAST against the set of all protein sequences with an experimental structure available in the Protein Data Bank (PDB). In all cases, multiple significant hits against sequences corresponding to HEGs of the LAGLIDADG family were identified (Fig. 2a). We then predicted the structure of the five HEGs with AlphaFold 3 (Abramson *et al.* 2024), and found that in all cases except one they presented the standard structure of double-motifs LAGLIDADG endonucleases (Fig 2b and c), differing only in the insertions present in the flexible loops connecting the core elements of the motifs. The remaining HEG instead was predicted to adopt a conformation of a single-motif LAGLIDADG HEG, which have previously been identified in other group I introns, and is therefore expected to function as a dimer (Lucas *et al.* 2001).

**Figure 3.**
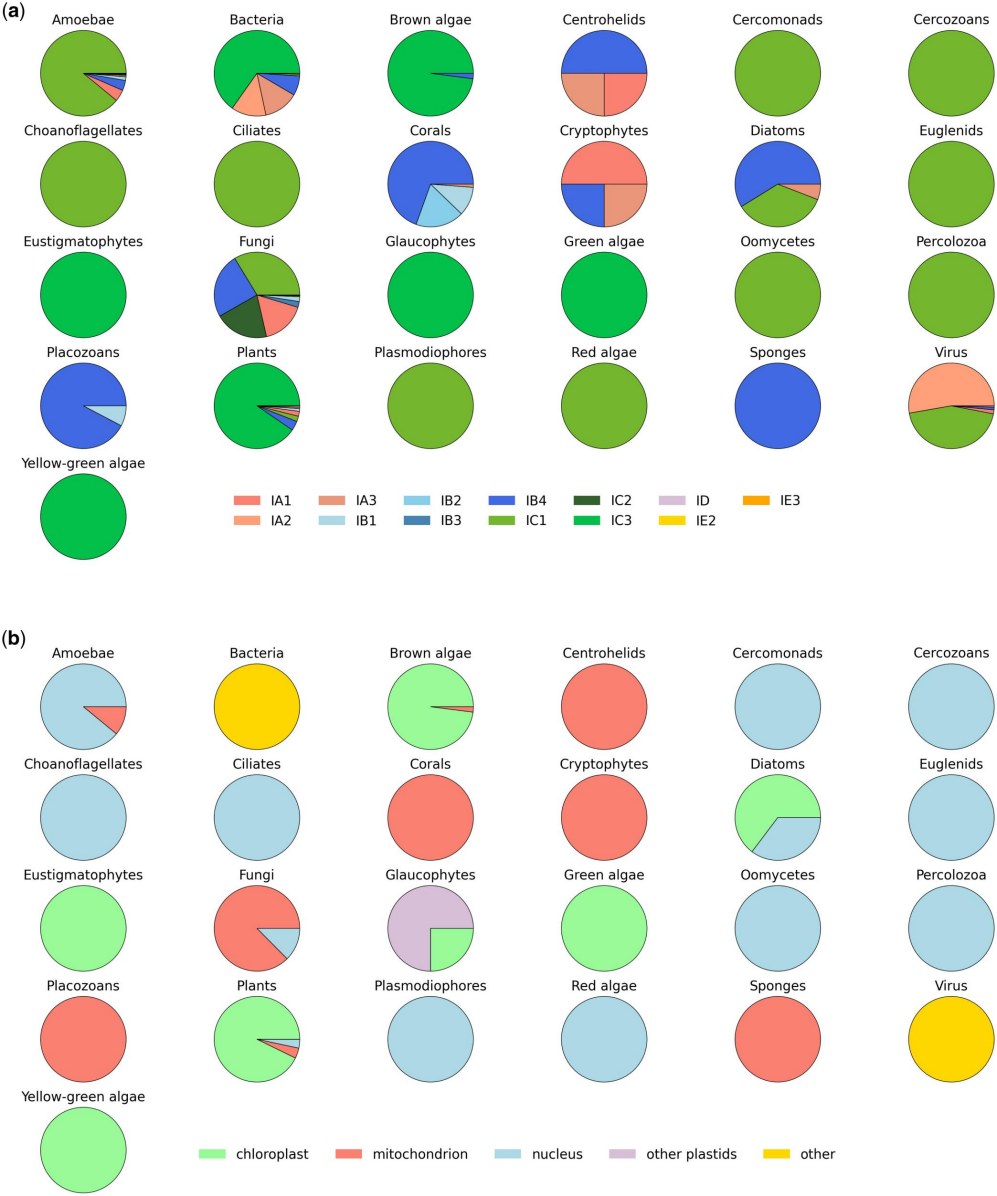
Distribution of introns in each organism type. (a) Distribution of intron subtypes. (b) Distribution of subcellular locations.

Analysis of the subtypes (Fig. 3a) and subcellular locations (Fig. 3b) assigned to the identified introns revealed that the largest population corresponds to IC3 introns present in plants, present mostly in chloroplasts. Fungi (the second largest source of group I introns), on the other hand, contained a variety of subtypes with considerable representation, with the four most frequent subtypes being IC1, IB4, IC2, and IA1. The only three multicellular animal organism types found to have group I introns (placozoans, corals, and sponges) all contained mostly mitochondrial introns of subtype IB4. The presence of mitochondrial introns of the same subtype in both placozoans and corals is in accordance with the previous hypothesis that Placozoa and Cnidaria are sister groups (Laumer *et al.* 2018). Interestingly, over 50% of viral group I introns belonged to subtype IA2, a subtype without significant presence in any other organism type except in bacteria, suggesting a potential horizontal transfer of group I introns between bacteriophages and their hosts.

**Figure 4.**
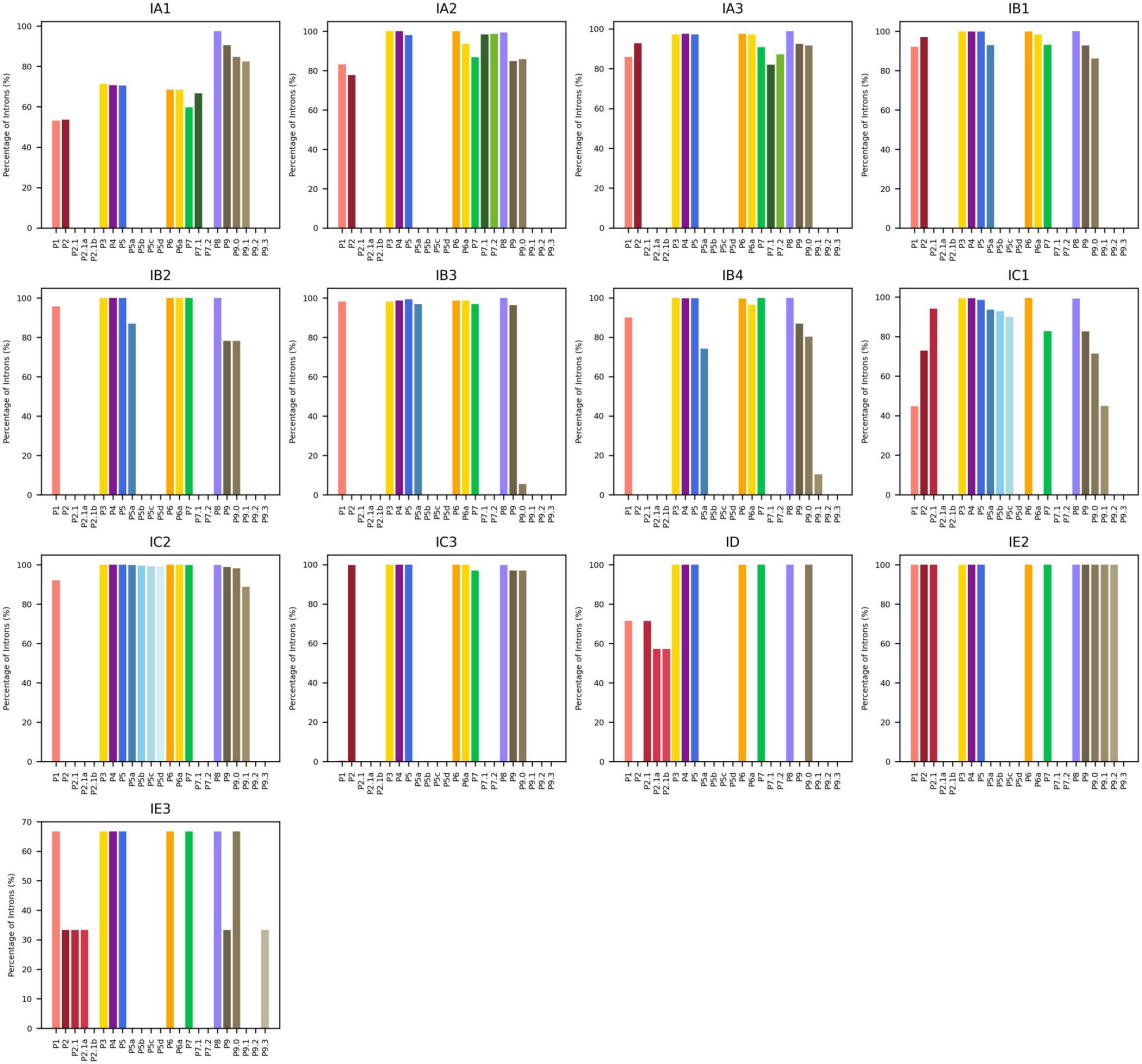
Presence of consensus structural elements across intron subtypes. Each bar plot corresponds to a subtype. Each bar represents the frequency of occurrence of a consensus structural elements across all of the introns of a given subtype.

**Figure 5.**
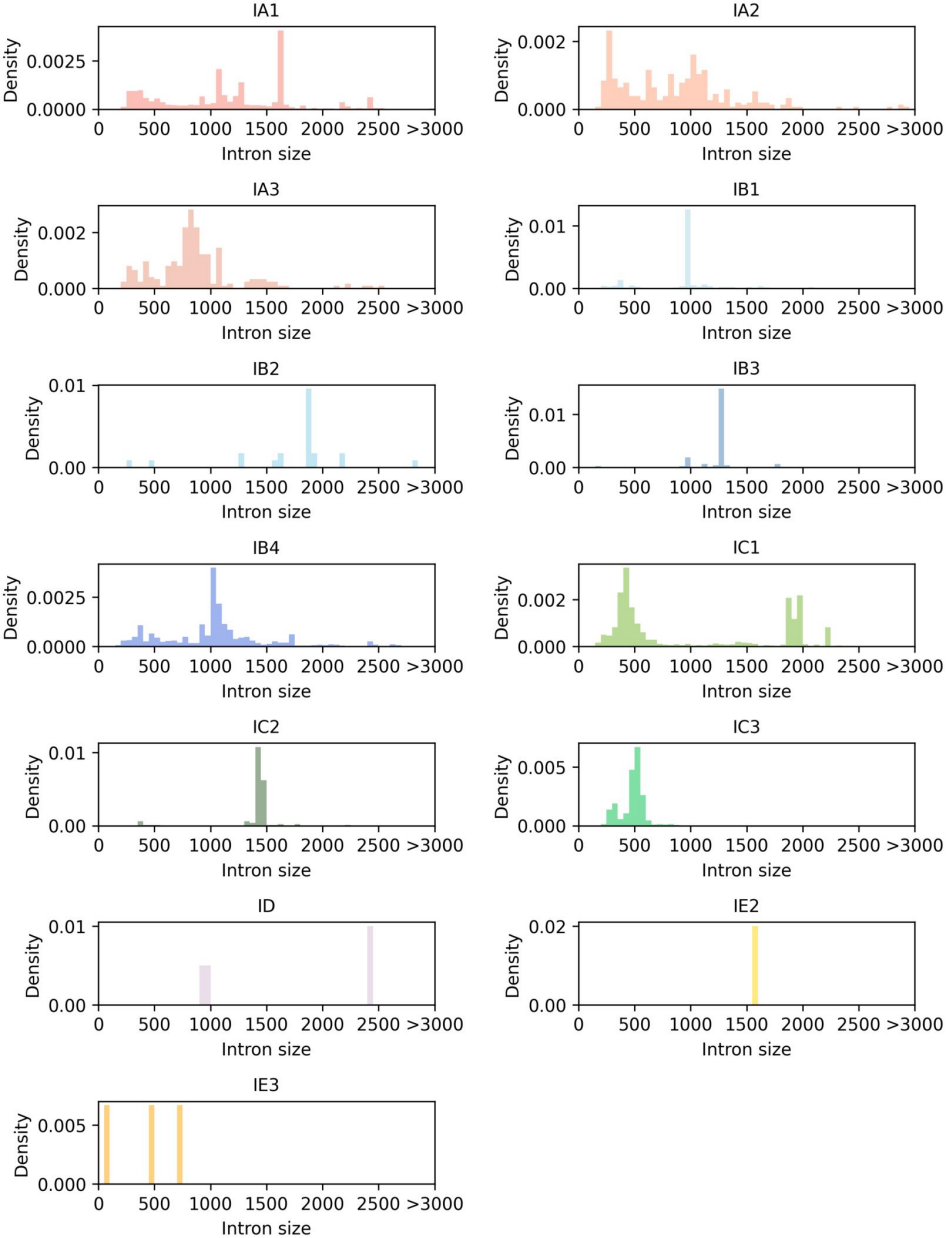
Distribution of intron sizes by subtype. Considerable differences in intron size distribution were observed among the different subtypes.

The majority of introns across all subtypes were also found to contain core structural elements critical for catalysis (Fig. 4). Generalized absences of specific elements in certain subtypes were observed, with differences even among closely related subtypes. For example, nearly all IC2 introns contain the P5(a-d) regions, but these are absent from IC3 subtype. We also analyzed the distribution of intron sizes across different intron subtypes, and found considerable differences (Fig. 5).

Thanks to the reliability of intron boundaries, the wide scope of the search performed here (covering the entire NCBI NT database), and the rich metadata added to each entry (including secondary structure information), we expect our database to constitute a valuable resource for studying and engineering group I introns. Additionally, we expect to provide periodic updates to our database, to account for the presence of group I introns in newly deposited sequences. The database, the code used to generate it and the multiple sequence alignments and CMs for each intron subtype are publicly available at https://github.com/LaraSellesVidal/Group1IntronDatabase, and base-pairing probability matrixes are available at https://www.ebi.ac.uk/biostudies/studies/S-BSST1399. A simple, self-contained GUI to facilitate preliminary exploration of the database is also available from the GitHub repository for all major platforms:

- macOS (Apple Silicon): intron_g1_DB_macOS_ARM64.app
- macOS (Intel): intron_g1_DB_macOS_x86_64.app
- Linux: intron_g1_DB_linux
- Windows: intron_g1_DB_windows.exe

**Figure 6.**
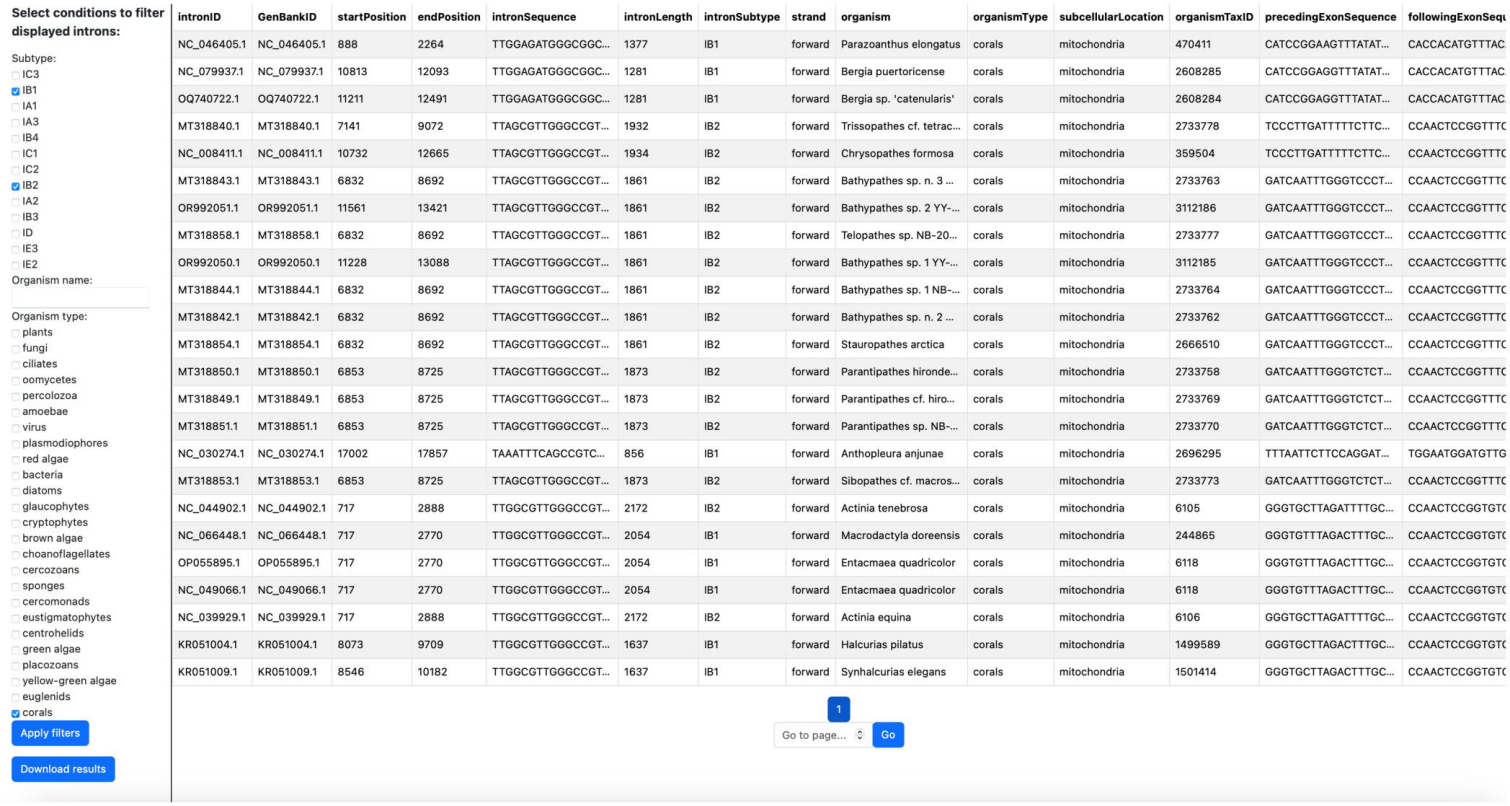
Graphical user interface for the group 1 intron database. The GUI is divided in two areas. The left panel allows to input selection criteria (intron subtype, organism name, and organism type), while the right panel displays the information for the group 1 introns fulfilling the selection criteria in a tabular format. The selected introns and all associated information can be downloaded into a single file with the *Download results* button. The contents of fields too large to be displayed in a single cell of reasonable size can be fully visualized by clicking on the corresponding cell.

The source code for the GUI is available at https://github.com/LaraSellesVidal/OnlineGroup1IntronDatabase. The database can also be accessed online at https://online-group-1-intron-database.onrender.com. The layout of the GUI is shown in Fig. 6. The GUI is divided into two areas: a left panel allowing selection of criteria to filter the shown group 1 introns, and a right panel displaying the group 1 introns that fulfil the input selection criteria. The right panel presents all the information about the selected introns in a tabular format. Available selection criteria are intron subtype, organism name, and organism type. The GUI also allows export of the selected introns directly into TSV files through the *Download results* button. This greatly simplifies selection of a subset of the available data for researchers interested in studying introns with specific features. For example, in Fig. 6, introns belonging to either the IB1 or the IB2 subtypes and located in corals are selected.

## 5 Conclusion

We have developed an up-to-date database of group I introns including rich annotations of multiple features. Furthermore, we provide, for the first time, a comprehensive set of putative homing endonucleases associated with the identified introns. Our workflow can be easily reproduced, and we therefore intend to provide regular updates of the database, as new releases of the NCBI NT database with new sequences become available, or additional data annotations are requested by the user community. We expect this actively maintained resource to be a valuable tool for the large-scale analysis and engineering of group I introns.

## Data Availability

The database, as well as the code to generate it and a GUI to facilitate its exploration, are available in GitHub at https://github.com/LaraSellesVidal/Group1IntronDatabase. The source code for the GUI implementation is available in GitHub at https://github.com/LaraSellesVidal/OnlineGroup1IntronDatabase. The database can also be accessed online at https://online-group-1-intron-database.onrender.com. Base-pairing probability matrixes are available separately in BioStudies at https://www.ebi.ac.uk/biostudies/studies/S-BSST1399.

## Appendix 1

Our database contains 29 fields for each intron, described below:

- intronID: a unique identifier for each intron, containing the GenBankID of the sequence in which it was found.
- GenBankID: the GenBankID of the sequence in which the intron was found.
- startPosition: the first position of the intron sequence, including the terminal U of the 5′ flanking exon.
- endPosition: the last position of the terminal sequence (most frequently, the terminal G).
- intronSequence: sequence of the intron.
- intronLength: length of the intron sequence.
- intronSubtype: subtype of the intron.
- strand: strand in which the intron was found (either *forward* or *reverse*).
- organism: species in which the intron is located.
- organismType: broad category to which the organism of the corresponding intron belongs.
- subcellularLocation: subcellular compartment where the sequence containing the intron is located.
- organismTaxID: unique NCBI taxonomy idenfier (*taxid*) of the organism of the corresponding intron.
- precedingExonSequence: sequence of the exon located before the intron (5′ exon), up to 300 nucleotides.
- followingExonSequence: sequence of the exon located after the intron (3′ exon), up to 300 nucleotides.
- eternafoldSecondaryStructure: EternaFold secondary structure prediction of the intron, in dot-bracket notation.
- viennaSecondaryStructure: ViennaRNA secondary structure prediction of the intron, in dot-bracket notation.
- contrafoldSecondaryStructure: CONTRAfold secondary structure prediction of the intron, in dot-bracket notation.
- rnastructureSecondaryStructure: RNAstructure secondary structure prediction of the intron, in dot-bracket notation.
- consensusSecondaryStructure: secondary structure prediction of the intron obtained by alignment to the group I intron covariance model with Infernal, in WUSS notation.
- consensusStructuralElements: group I intron structural element assignment based on alignment to the corresponding group I intron subtype covariance model with Infernal.
- infernalHitStartPosition: the first position of the region in the containing GenBank sequence against which a hit with the group I intron covariance model was found.
- infernalHitEndPosition: the last position of the region in the containing GenBank sequence against which a hit with the group I intron covariance model was found.
- infernalHitEValue: e-value of the hit with the group I intron covariance model.
- infernalHitBitScore: bit score of the hit with the group I intron covariance model.
- infernalHitU10Position: position in the containing GenBank sequence that was aligned with U10 of the group I intron covariance model (note that, in some cases, no position was aligned with U10).
- infernalSubtypeHitStartPosition: the first position of the region in the containing GenBank sequence that aligned with the covariance model of the corresponding intron subtype.
- infernalSubtypeHitStartPosition: the last position of the region in the containing GenBank sequence that aligned with the covariance model of the corresponding intron subtype.
- infernalSubtypeHitEValue: e-value of the hit with the corresponding intron subtype covariance model.
- infernalSubtypeHitBitScore: bit score of the hit with the corresponding intron subtype covariance model.

## Appendix 2

We have found that Microsoft Excel can mishandle the database file when opening it due to not recognizing the file encoding as UTF-8. In order to ensure all fields are properly read in Microsoft Excel, the following steps should be followed:

1. Go to Data tab, Get Data submenu and select From Text, then choose the database file.
2. In the next screen, options must be selected as Delimited file (since columns are not fixed width) and, importantly, Unicode (UTF-8) in the File origin menu.
3. Then, Tab should be selected as the delimiter.
4. In the final screen, the data type of specific columns can be chosen. We recommend choosing explicitly type Text for columns *precedingExonSequence* and *consensusStructuralElements* to avoid Excel interpreting them as some other data type.
